# Supplementary material for: Splice-Junction-Based Mapping of Alternative Isoforms in the Human Proteome
Source: Cell Rep. Author manuscript; Available in PMC 2020 Jan 15. (PMC6961840; doi:10.1016/j.celrep.2019.11.026)
Supplement: 2 [file NIHMS1546469-supplement-2.zip › DF1/PXD010154/Colon/Colon_12_IDH3G_LSWHPWTMRMLHSYATSIR.pdf]

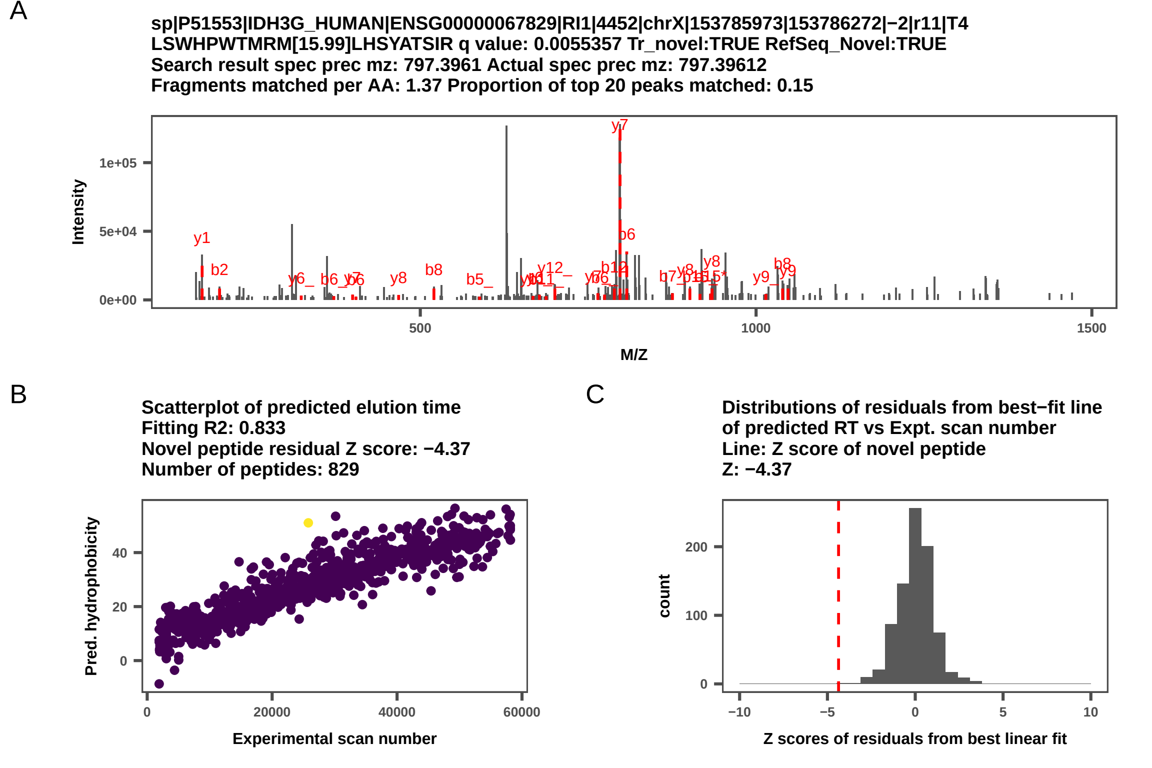
A
sp|P51553|IDH3G_HUMAN|ENSG00000067829|RI1|4452|chrX|153785973|153786272|−2|r11|T4
LSWHPWTMRM[15.99]LHSYATSIR q value: 0.0055357 Tr_novel:TRUE RefSeq_Novel:TRUE
Search result spec prec mz: 797.3961 Actual spec prec mz: 797.39612
Fragments matched per AA: 1.37 Proportion of top 20 peaks matched: 0.15
Intensity
1e+05
5e+04
0e+00
M/Z
500
1000
1500
y7
y1
b2
b6
b8
y8
B
Scatterplot of predicted elution time
Fitting R2: 0.833
Novel peptide residual Z score: −4.37
Number of peptides: 829
Pred. hydrophobicity
40
20
0
0
20000
40000
60000
Experimental scan number
C
Distributions of residuals from best−fit line
of predicted RT vs Expt. scan number
Line: Z score of novel peptide
Z: −4.37
count
200
100
0
−10
−5
0
5
10
Z scores of residuals from best linear fit
